# Long Non‐Coding RNA LINC01116 Promotes the Proliferation of Lung Adenocarcinoma by Targeting miR‐9‐5p/CCNE1 Axis

**DOI:** 10.1111/jcmm.70270

**Published:** 2024-12-08

**Authors:** Hui Zhang, Wenwen Cai, Yiyan Miao, Yihang Gu, Xiaorong Zhou, Hiroyasu Kaneda, Lan Wang

**Affiliations:** ^1^ The Jiangyin Clinical College of Xuzhou Medical University Xuzhou China; ^2^ Sanmen County People's Hospital Taizhou China; ^3^ Department of Geriatrics The Jiangyin Clinical College of Xuzhou Medical University Jiangyin China; ^4^ Department of Immunology, School of Medicine Nantong University Nantong China; ^5^ Department of Clinical Oncology, Graduate School of Medicine Osaka Metropolitan University Osaka Japan; ^6^ Department of Respiratory and Critical Care Medicine The Jiangyin Clinical College of Xuzhou Medical University Jiangyin China

**Keywords:** CCNE1, cell proliferation, LINC01116, LUAD, miR‐9‐5p

## Abstract

Long non‐coding RNA (lncRNA) LINC01116 is crucial in promoting cell proliferation, invasion and migration in solid tumours, including lung adenocarcinoma (LUAD). LINC01116 acts as a competing endogenous RNAs (ceRNA) that binds competitively to microRNAs and plays a critical role in tumour migration and invasion. However, other mechanisms of action besides the ceRNA theory have been rarely reported and remain to be elucidated further. The differences in RNA and protein levels in cells and tissues were assessed through real‐time quantitative PCR and Western blot analysis. In vitro functional assays and in vivo xenograft models were used to analyse the function of LINC01116 in LUAD. Thus, the molecular correlation between miR‐9‐5p and CCNE1 was investigated through direct and indirect mechanism experiments. LINC01116, miR‐9‐5p and CCNE1 were upregulated in LUAD cell lines and tissues and were associated with a poor prognosis in patients. LINC01116 depletion inhibited proliferation but facilitated cell apoptosis. AGO2‐RNA binding protein immunoprecipitation (AGO2‐RIP) experiments confirmed that AGO2 binds to LINC01116 and miR‐9‐5p, indicating that LINC01116 interacts with miR‐9‐5p. The overexpression of miR‐9‐5p and CCNE1 effectively counteracts the biological effects of LINC01116 knockdown on reduced proliferation and cell cycle arrest in LUAD cells. The downregulation of miR‐9‐5p significantly reduces the CCNE1 level in A549 cells, and the upregulation of LINC01116 counteracts the downregulation of miR‐9‐5p effect, restoring the expression level of CCNE1. Our data demonstrated that LINC01116 regulates the expression of CCNE1 by positively regulating miR‐9‐5p, thereby affecting cell cycle, proliferation and participating in the development of LUAD.

## 1 Introduction

Lung cancer is the most commonly diagnosed cancer worldwide. The disease accounts for 11.6% of the total cancer cases and 18.4% of the total cancer mortality [1]. Non‐small cell lung cancer (NSCLC) accounts for approximately 85% of the total number of lung cancers, while lung adenocarcinoma (LUAD), the main histological subtype of lung cancer, accounts for approximately 40% of lung cancer cases [2]. LUAD has gradually replaced squamous cell carcinoma and has become the most common pathological type of lung cancer [3]. It exhibits complex biological characteristics with a high degree of malignancy, leading to the fact that most patients are in the advanced stage at the time of diagnosis and have metastasis and poor prognosis [4]. In the past 30 years, although significant progress has been made in the treatment of lung cancer, the survival rate of patients with advanced (stage IV) lung cancer is not optimistic, and the 5‐year survival rate is only 9%, while the 5‐year survival rate of patients with early (stage IA) lung cancer is > 77% [5]. Early LUAD may not be able to distinguish between invasive and non‐invasive types by chest computed tomography (CT). Therefore, improving the curative effect of the comprehensive treatment of lung cancer, inhibiting malignant proliferation, invasion and metastasis of early tumour, and enhancing the prognosis of patients by exploring the unidentified biomarkers associated with LUAD are beneficial for individualised treatment strategies according to molecular typing.

Long non‐coding RNA (lncRNA) is transcribed from polymerase II (Pol II), with a length of > 200 nucleotides and cannot encode proteins [6]. Several studies have shown that the abnormal expression of lncRNA as a regulatory molecule plays a key role in the pathophysiological processes of malignant proliferation, differentiation, apoptosis and migration of tumours, which has become a hotspot in tumour research [7]. A large number of studies have shown a new regulatory circuitry called competing endogenous RNAs (ceRNAs), in which lncRNAs competitively bind microRNAs (miRNAs), participate in the regulation of coding genes at the post‐transcriptional level and inhibit the restriction or degradation of the translation level caused by the combination of miRNAs and messenger RNA (mRNA) to maintain the stability of target genes and the protein translation [8]. For example, LINC‐MD1 regulates the expression of transcription factors *MAML1* and *MEF2C*, activates specific gene expression of muscle and shortens the differentiation time of human myoblasts by competitively binding with *miR‐133* and *miR‐135* [9]. The expression of LINC00973 is significantly elevated in NSCLC and is associated with poor patient prognosis [10]. Long gene non‐protein coding RNA 01116 (LINC01116) is a lncRNA, abnormally expressed in various cancers, including lung cancer, gastric cancer, colorectal cancer, glioma and osteosarcoma, which plays a crucial role in promoting cell proliferation, invasion, migration and apoptosis [11]. Another study indicated that LINC01116 is associated with cell senescence and is closely related to apoptosis; thus, it can predict the prognosis of LUAD patients and tumour immune microenvironment [12]. Additionally, recent studies [13] have shown that low LINC01116 expression is generally diagnosed with TNM I rather than TNM I/III, suggesting its involvement in lung cancer metastasis and invasiveness. Thus, LINC01116 plays a key role in tumour progression and may serve as a valuable diagnostic and prognostic marker for lung cancer. Next, we analysed the biological information database and confirmed that LINC01116 was upregulated in LUAD cells and could serve as a critical regulatory factor for the development of LUAD.

Previous studies indicate that non‐coding RNAs, including lncRNAs and miRNAs, play roles in cell cycle regulation [14]. Despite advances in genomic technology and analytics, identifying new lncRNA‐related ceRNA networks that impact the cell cycle and LUAD remains challenging. Therefore, in the present study, we aimed to clarify the role of LINC01116 in the development and its possible regulatory mechanism in the malignant behaviour of LUAD. The results showed that LINC01116 is overexpressed in LUAD and is associated with poor prognosis. The gene co‐expression network revealed that LINC01116 binds to miR‐9‐5p, and the target of LINC01116 and miR‐9‐5p could be predicted by the starBase database. Similarly, the analysis of the biological database showed that cyclin 1‐CCNE1, as a candidate target gene complex downstream of miR‐9‐5p, was positively regulated by miR‐9‐5p. Several experiments indicated that LINC01116 positively regulated the expression of CCNE1 by targeting miR‐9‐5p. The downregulation of LINC01116 inhibits the proliferation and promotes the apoptosis of LUAD cells. This study builds on prior research by detailing direct and indirect regulatory pathways involving LINC01116 and miR‐9‐5p, offering potential strategies for combined therapies. In short, the investigation of its function and mechanism, in combination with clinical information analysis, suggested that LINC01116 is a potential diagnostic biomarker and therapeutic target for LUAD.

## 2 Materials and Methods

### 2.1 Target Prediction

The clinical information and corresponding sequencing data of 594 patients with LUAD were obtained from https://portal.gdc.cancer.gov/TCGA. The present study comprised 594 cases, including 535 LUAD tumours and 59 cases of LUAD para‐cancerous tissue samples. After obtaining the expression profile data and clinical data of lncRNAs, miRNAs and mRNAs, the differentially expressed genes were screened using the ‘edgeR’ differential analysis data package of R3.6.0 software; the screening conditions were Log2FC(T/N) ≥ 2 or ≤ −2 and *p* < 0.05. The ‘WGCNA’ data package was used for network analysis of weighted gene co‐expression combined with clinical data for survival analysis and starBase V3.0 database for target gene prediction.

### 2.2 Patients and Clinical Samples

Fresh LUAD clinical specimens and paired para‐cancerous tissue samples were obtained from patients undergoing surgical resection between 2015 and 2020 at Jiangyin People's Hospital. All cases were diagnosed by clinical, imaging and pathological approaches, and adjuvant therapy was not administered before the operation. The current study was performed in accordance with the Helsinki Declaration. Written informed consent was obtained from all participants.

### 2.3 Cell Line and Culture

Lung cancer cell lines (A549, H441, pC9 and H358) and normal lung epithelial cells (BEAS‐2B) were obtained from the Center for Excellence in Molecular Cell Science (Shanghai Institute of Biochemistry and Cell Biology, Shanghai, China). The cells were routinely cultured in RPMI1640 medium (Gibco, USA), supplemented with 10% foetal bovine serum (FBS, Gibco, USA) and 1% penicillin–streptomycin (Gibco, USA) in 5% CO_2_ at 37°C.

### 2.4 Cell Transfection

LINC01116 knockdown A549 cells were transfected with the packaged recombinant lentivirus. sh‐LINC01116 and sh‐*NC* virus solution were procured from Hanheng Biotechnology (Shanghai, China). The cells in the logarithmic growth phase were collected and inoculated into a six‐well plate at a density of 5 × 10^5^/well in 2 mL of complete medium and cultured under 5% CO_2_ at 37°C for 24 h. Lentiviral transfection can be carried out when the cell density reaches a 70%–90% confluency, and the transfectants are screened under puromycin selection after 48 h. In addition, pcDNA3.1‐LINC01116, pcDNA3.1‐CCNE1, pcDNA3.1‐miR‐9‐5p and negative control pcDNA3.1, as well as the inhibitor‐miR‐9‐5p, si‐LINC01116 and inhibitor‐NC, were synthesised by GenePharma Company (Shanghai, China) and transfected into A549 cells for 48 h using the ExFect Transfection Reagent (Vazyme Biotechnology, Nanjing, China).

### 2.5 Quantitative Real‐Time Polymerase Chain Reaction (qRT‐PCR)

Total RNA was extracted using TRIzol Reagent (Thermo Fisher, USA) and reverse‐transcribed into cDNA with the HiScript III 1st Strand cDNA Synthesis Kit (+gDNA wiper) (Vazyme Biotechnology, Nanjing, China) in an SYBR qPCR master mix for qRT‐PCR detection. *GAPDH* was selected as the internal parameter, and the relative expression was evaluated by calculating the 2^−ΔΔCT^ value. The primers were synthesised by Sangon (Shanghai, China).

### 2.6 Western Blot

Total protein was extracted with radioimmunoprecipitation assay (RIPA) lysis buffer (Beyotime, Shanghai, China) on ice, and the whole protein was analysed and quantified with the Bradford assay (Bio‐Rad Laboratories, Hercules, CA, USA). Then, each target protein (CCNE1, Ki67, PCNA, p16, p53, MCM7) was separated by SDS‐polyacrylamide gel (CellorLab, Shanghai, China) and transferred to polyvinylidene fluoride (PVDF, Cytiva, Germany), using tris buffer (Absin, Shanghai, China). PVDF was placed in a 5% skimmed milk (Beyotime, Shanghai, China) for 2 h and probed with primary antibodies, such as anti‐GAPDH (Proteintech, Cat No. 60004‐1‐Ig, Wuhan, China), anti‐CCNE1 (Proteintech, Cat No. 11554‐1‐AP, Wuhan, China), anti‐Ki67 (Abcam, ab15580), anti‐PCNA (Cell Signalling Technology, ab29, USA), anti‐p16 (Cell Signalling Technology, 18769, USA), anti‐p53 (Cell Signalling Technology, 2527, USA) and anti‐MCM7 (Cell Signalling Technology, 3735, USA) overnight at 4°C. Then, the membranes were incubated with the appropriate secondary antibody anti‐rabbit IgG‐HRP (Abcam, ab7090, UK) or anti‐mouse IgG‐HRP (Abcam, ab7068, UK) at 37°C for 1 h. Finally, the immunoreactive bands were detected by an enhanced chemiluminescence (ECL) detection kit (Vazyme Biotechnology, e411, Nanjing, China).

### 2.7 Cell Proliferation Assay

Cell proliferation was evaluated using a cell counting kit (CCK‐8 kit, KGA317, KeyGen Biotech, Nanjing, China) and a BeyoClick EdU Cell Proliferation Kit with Alexa Fluor 488 (RiboBio, Guangzhou, China). For CCK‐8 detection, the transfected cells (3 × 10^3^) were seeded into 96‐well plates and cultured for 24, 48 and 72 h. Then, 100 mL of CCK‐8 reagent was added to each well and incubated at 37°C for 2 h in the dark. The absorbance of the reaction was measured at 450 nm on a microplate reader. The growth curves of the cell line were drawn based on the results of the CCK‐8 assay. For 5‐ethynyl‐2′‐deoxyuridine (EdU) assay, the transfected cells (1 × 10^5^) were seeded into 24‐well plates and cultured for 24 h. Subsequently, 200 mL of EdU cell medium was supplemented to each well, and the reaction was incubated for 2–3 h. Then, the cells were fixed with 4% paraformaldehyde at 37°C for 30 min and permeabilised in 0.5% Triton X‐100. After nuclear staining with Hoechst 33342 in the dark at 37°C for 30 min, cell proliferation was observed under a fluorescence microscope. The number of cells was calculated and analysed by Image J software.

### 2.8 Flow Cytometry

Apoptosis was detected using an Annexin V‐FITC/PI detection kit (Vazyme Biotech, A211, Nanjing, China), and cell cycle was determined with a Cell Cycle Detection Kit (KeyGEN Biotech, KGA511, Nanjing, China). The cell precipitates were collected to detect early apoptosis using Annexin V‐FITC and late apoptosis by propidium iodide (PI). Flow cytometry (CytoFLEX S, Beckman Coulter, Suzhou, China) was applied for the detection of cell apoptosis, and FlowJo V10 was utilised to calculate the apoptotic cell number. Moreover, the cell cycle was detected by flow cytometry, and the red fluorescent cells were analysed at 488 nm.

### 2.9 Fluorescence In Situ Hybridisation (FISH)

LINC01116 specific probe was synthesised by RiboBio Co. Ltd. The denatured probe was hybridised with a glass specimen, which had been degenerated, dehydrated at 37°C, and hybridised overnight. After the natural drying of the slide, DAPI staining was performed, and the stained cells were analysed under a fluorescence microscope.

### 2.10 AGO2‐RNA Binding Protein Immunoprecipitation (AGO2‐RIP)

Magna RIP RNA Binding Protein Immunoprecision Kit (Millipore, USA) was employed. After processing the sample and magnetic bead pretreatment, the target protein antibody (anti‐AGO2) was added to the pre‐treated RIP tube; also, the IgG control group was set up. After RNA extraction, qRT‐PCR was adopted to detect RNA enrichment using target gene primers (LINC01116 and miR‐9‐5p).

### 2.11 Xenograft Tumour Experiment

BALB/c nude mice were purchased from Beijing Vital River Laboratory Animal Technology Co. Ltd. All animal‐associated protocols were conducted in accordance with the standards of Animal Experiment Ethics Committee of the Experimental Center of Nantong University (Jiangsu, China). Transfected A549 cells (3 × 10^5^) were injected subcutaneously into 6‐week‐old mice, and the tumour volumes were measured every 3 days. After 28 days, the mice were sacrificed; the tumours were excised, weighed and the volume measured.

### 2.12 Statistical Analysis

The experimental data were processed using GraphPad Prism 6.0 and Image J software. Student's *t*‐test and one‐way analysis of variance (ANOVA) were used in this study. All cell assays were repeated three times, and each replicate consisted of three animals per group for the model. Data were expressed as mean ± standard deviation (SD), and *p* < 0.05 indicated statistical significance.

## 3 Results

### 3.1 Differential Expression of lncRNAs, miRNAs and mRNAs in TCGA Database

**FIGURE 1:**
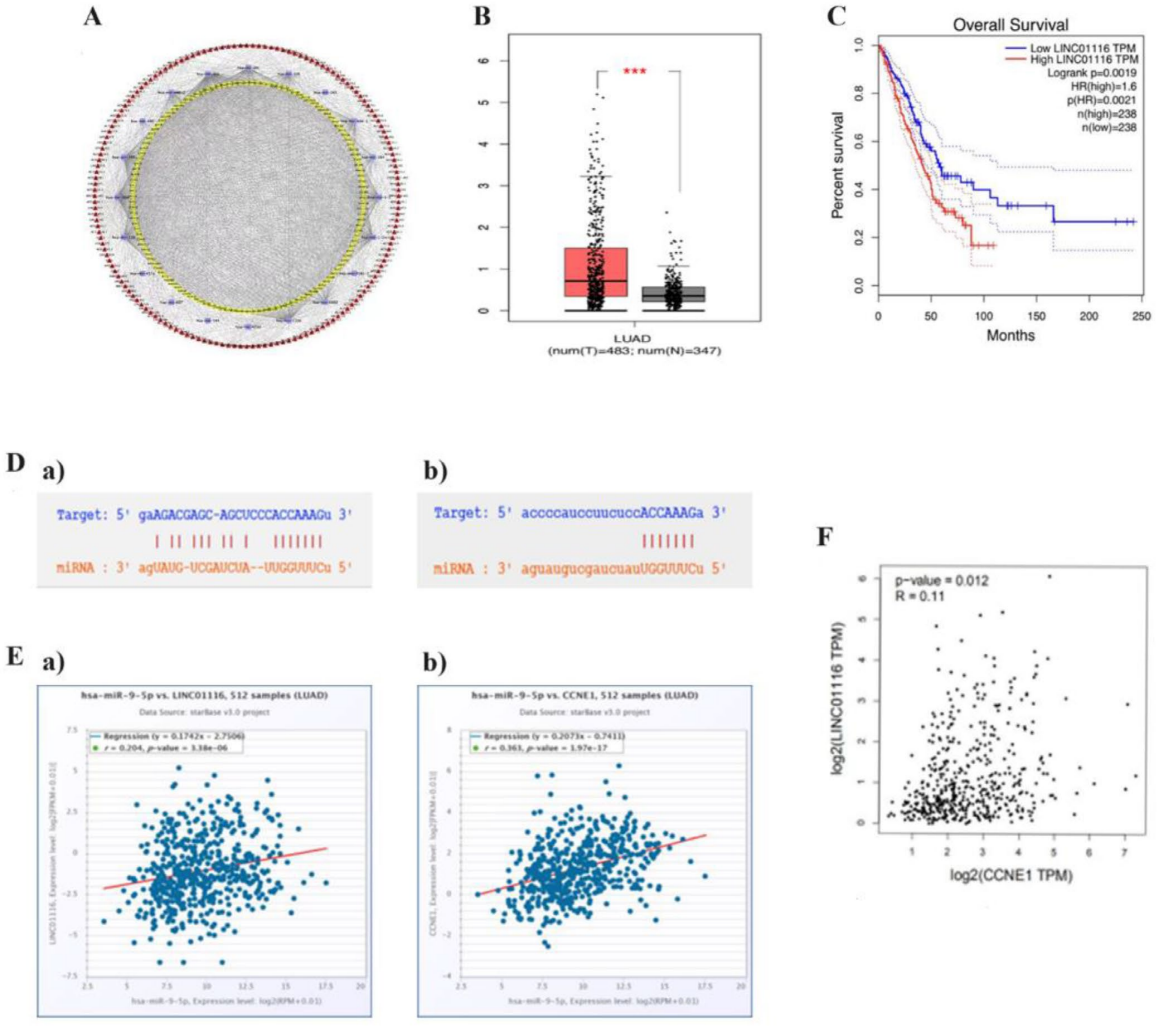
(A) The ceRNA network of the co‐expression of lncRNAs, miRNAs and mRNAs was obtained by WGCNA analysis. (B) Expression of LINC01116 in LUAD and normal tissue samples. The expression level of LINC01116 in LUAD samples was significantly higher than that in normal tissues (***p < 0.001). (C) Kaplan–Meier survival analysis was applied to show significantly different survival curves between LUAD patients with high and low expression levels of LINC01116 (*p* = 0.0019). The abscissa of the survival curve is the observation time, and the ordinate is the overall survival rate. (D. a) Hypothetical binding site of LINC01116 and miR‐9‐5p. (b). starBase database predicted the positive correlation between LINC01116 and miR‐9‐5p (*p* = 3.38e‐06). (E. a) Hypothetical binding site of miR‐9‐5p and CCNE1. (b) starBase database predicted the positive correlation between LINC01116 and miR‐9‐5p (*p* = 1.97e‐17). (F) starBase database predicted the positive correlation between LINC01116 and CCNE1 (*p* = 0.012).

The microarray analysis of the expression profile and clinical data of lncRNA identified the miRNA and mRNA of LUAD from TCGA, following which we screened out the differentially expressed lncRNAs, miRNAs and mRNAs. A total of 1616 lncRNAs (1410 lncRNAs with high expression and 206 lncRNAs with low expression), 134 miRNAs (114 miRNAs with high expression and 20 miRNAs with low expression) and 2483 mRNA (1956 mRNA with high expression and 527 mRNA with low expression) were differentially expressed. According to the co‐expression network of ceRNA, 20 lncRNAs with significant differences in survival analysis and prognosis were screened: AC012213.4, AC022784.1, AC034223.1, AC068189.1, AL139393.2, AL353746.1, AL355596.1, AL512363.1, AP005137.2, ITGB1‐DT, LINC00518, LINC01116, LINC01312, LINC01711, LINC01775, LINC02310, LINC02535, SATB2‐AS1, SCAT1 and SYNPR‐AS1, respectively (Figure 1A). The ‘edgeR’ and ‘WGCNA’ data packets of R3.6.0 software, combined with the molecules in the network and clinical data, were used for the expression of LINC01116 in LUAD and normal tissue samples. The expression level of LINC01116 in LUAD samples was significantly higher than that in normal tissues (***p < 0.001) (Figure 1B). These data packets were also used for survival analysis; the expression level of LINC01116 was significantly higher in LUAD samples than in normal tissues and also showed significant difference in the survival curves of LUAD patients with high and low expression levels of LINC01116 (*p* = 0.0019) (Figure 1C). Finally, LINC01116 was confirmed as a major regulatory factor for the occurrence and development of LUAD. According to the gene co‐expression network, putative binding sites were identified between miR‐9‐5p and LINC01116 (Figure 1Da), and miR‐9‐5p could combine multiple genes. The KEGG enrichment analysis revealed that the target genes were closely related to the cell cycle. Strikingly, CCNE1, a marker of cell cycle, was selected from among the candidate target genes through further screening by starBase V3.0 (Figure 1Db). Meanwhile, the analysis using the starBase V3.0 database showed a positive correlation between LINC01116 and the expression level of miR‐9‐5p and CCNE1 (Figure 1E), indicating that LINC01116 can positively regulate CCNE1 (Figure 1F). The KEGG pathway analysis suggested that the PI3K‐Akt signalling pathway and its downstream cell cycle, endocytosis and the related p53 signalling pathway were highly correlated with the downregulation of LINC01116, *CDK6* and *MDM2* and were differentially expressed in both the metabolic pathways. MDM2 is the most effective negative regulator of p53, and the activity of cyclin‐CDK6 kinase is inhibited by p16 [a member of cyclin‐dependent kinase inhibitor (CDKI) INK4 family]. Therefore, p53 and p16 were considered as the targets of subsequent studies. Therefore, we speculated that LINC01116 positively regulates the expression of CCNE1 through targeting miR‐9‐5p, thereby affecting cell cycle and apoptosis.

### 3.2 High Expression of LINC01116, CCNE1 and miR‐9‐5p in LUAD

**FIGURE 2:**
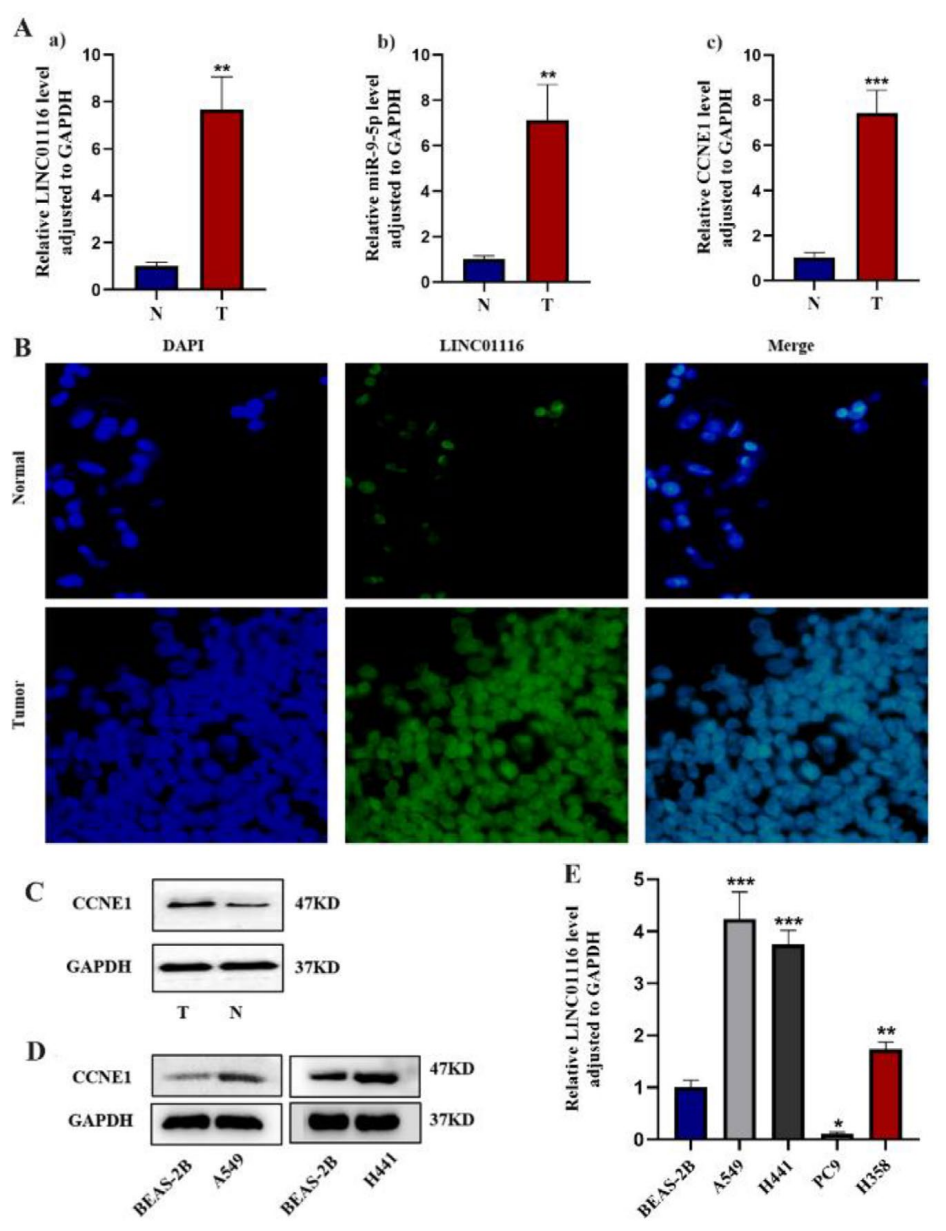
(A) The expression level of LINC01116, miR‐9‐5p and CCNE1 in tumour tissue (T) was analysed by qRT‐PCR [**p < 0.01, ***p < 0.001 vs. normal tissue (N)]. (B) Sub‐cellular localization of LINC01116 by FISH assays and its statistical analysis (**p < 0.01). (C) Protein expression of CCNE1 in tumour tissue measured by the Western blot analysis (*p < 0.05 vs. normal tissue). (D) Protein expression of CCNE1 in A549 or H441 cells measured by the Western blot analysis. (E) Expression of LINC01116 in various cell lines was quantitatively assessed via qRT‐PCR (*p < 0.05, **p < 0.01, ****p < 0.0001 vs. BEAS‐2B).

In order to verify the accuracy of the prediction, we collected 10 cases of LUAD clinical specimens and set up LUAD tissue (T) and matched para‐cancerous tissue (N). qRT‐PCR results showed that the expression levels of LINC01116, miR‐9‐5p and CCNE1 were significantly higher in LUAD tumour tissues than in the corresponding adjacent tissues, which was consistent with the results of the previous database analysis (Figure 2A). FISH assays demonstrated that LINC01116 was localised in the nucleus, and the expression was significantly increased in LUAD tissues (Figure 2B). The results of the Western blot showed that the expression level of CCNE1 was increased in LUAD tissues with a high expression of LINC01116 (Figure 2C).

Subsequently, we chose the normal lung epithelial cell line BEAS‐2B as the control group, lung cancer cell lines with a high expression of LINC01116, and finally A549 and H441 cells for the follow‐up experiment (Figure 2E). The results of the Western blot further confirmed that the expression of CCNE1 was upregulated in LUAD cells (Figure 2D), indicating that LINC01116, miR‐9‐5p and CCNE1 are upregulated in LUAD.

### 3.3 Silencing LINC01116 Inhibits the Proliferation of LUAD Cells In Vitro While Promoting Cell Apoptosis

**FIGURE 3:**
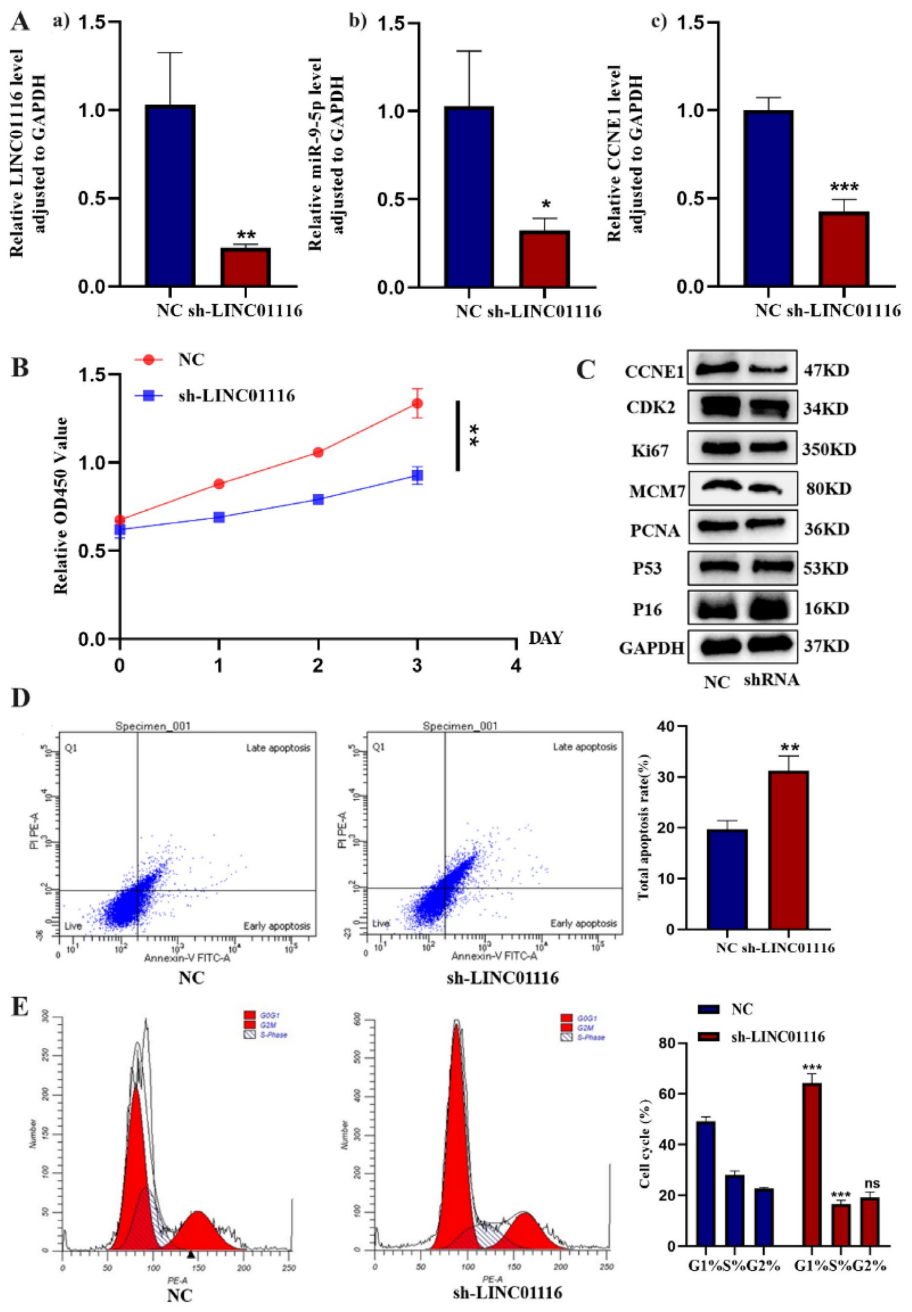
(A: a). Silencing efficiency of sh‐LINC01116 in A549 cells (**p < 0.01). (b and c). Relative expression level of miR‐9‐5p and CCNE1 in the sh‐LINC01116 group was assessed by qRT‐PCR analysis (*p < 0.05, **p < 0.01, ***p < 0.001 vs. NC group). (B) CCK‐8 assay showed that LINC01116 stable knockdown inhibited the proliferation of A549 cells. (C) Protein levels of CCNE1, CDK2, Ki‐67, MCM7, PCNA, p53 and p16 in the sh‐LINC01116 group, as measured by the Western blot. (D) Apoptosis rate of A549 cells, treated with negative control and LINC01116 shRNA (**p < 0.01). (E) Cell cycle of A549 cells, treated with negative control and LINC01116 shRNA, was analysed by flow cytometry (***p < 0.001).

In order to study the regulatory effect of LINC01116 on the cell biological behaviour, we transfected sh‐LINC 01116 and sh‐NC into A549. The expression of LINC01116 was downregulated in the sh‐LINC01116 group (vs. sh‐NC group), indicating a satisfactory transfection effect (Figure 3Aa). qRT‐PCR results showed that the expression of miR‐9‐5p and CCNE1 decreased in the knockdown group (Figure 3Ab,c). In addition, the results of the CCK‐8 cell proliferation demonstrated that silencing LINC01116 by shRNA significantly inhibits the proliferation of LUAD cells (Figure 3B). The results of the Western blot showed that compared to NC, the levels of cyclin CCNE1, CDK2 and proliferation markers (Ki‐67, MCM7 and PCNA) were decreased, while the levels of cell senescence markers (p53 and p16) increased after the downregulation of LINC01116 (Figure 3C). Flow cytometry results suggested that the apoptotic rate of A549 cells transfected with sh‐LINC01116 was higher than that of NC group (**p < 0.01) (Figure 3D), which was further confirmed by the Western blot, suggesting that the downregulation of LINC01116 induces apoptosis (Figure 3C). In addition, the proportion of sh‐NC‐ and sh‐LINC01116‐treated cells was increased at the G0/G1 phase (***p < 0.01 vs. NC group) but was decreased at the S phase (***p < 0.01 vs. NC group), respectively, which might inhibit cell division (Figure 3E). Therefore, we speculated that LINC01116 promotes the proliferation and inhibits the apoptosis of LUAD cells in vitro.

### 3.4 Overexpression of miR‐9‐5p or CCNE1 Promotes the Proliferation of LUAD Cells In Vitro While Inhibiting Cell Apoptosis

**FIGURE 4:**
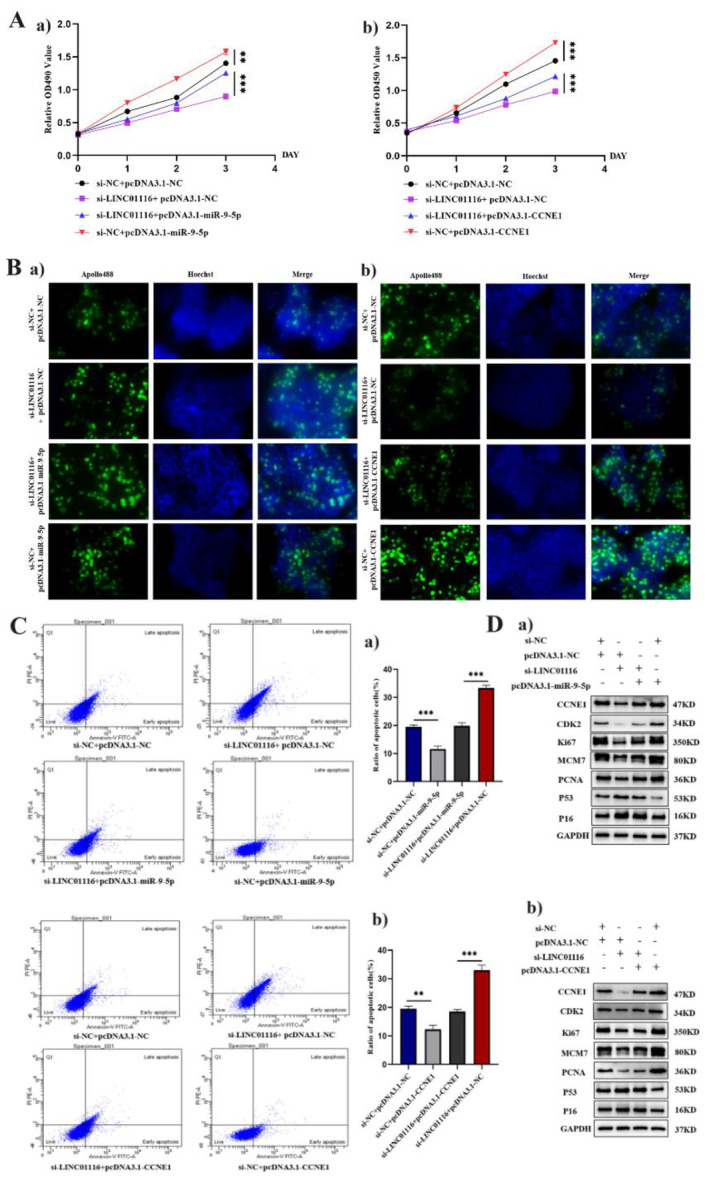
(A) CCK‐8 assay showed that LINC01116 stable knockdown inhibited the growth of A549 cells, and the overexpression of miR‐9‐5p or CCNE1 promoted A549 cell growth (***p < 0.001). (B) EdU assays were employed to assess the cell proliferation capacity after the downregulation of LINC01116 and upregulation of miR‐9‐5p or CCNE1. (C) After treatment with negative control, si‐LINC01116 or pcDNA3.1‐miR‐9‐5p/pcDNA3.1‐CCNE1 plasmid, A549 cells were stained and analysed by flow cytometry (***p < 0.001). (D) Protein expression of CCNE1, CDK2, Ki‐67, MCM7, PCNA, p53 and p16 in the si‐LINC01116 and pcDNA3.1‐miR‐9‐5p groups or pcDNA3.1‐CCNE1 group, as measured by the Western blot.

Next, we assessed the potential effects of miR‐9‐5p and CCNE1 on cell proliferation, apoptosis and cell cycle in LUAD cells with a high expression of LINC01116 separately. LUAD cells were co‐transfected with pcDNA3.1‐miR‐9‐5p, si‐LINC01116 or pcDNA3.1‐CCNE1, si‐LINC01116. The results of cell proliferation assays, CCK‐8 and EdU, showed that the overexpression of miR‐9‐5p or CCNE1 promotes cell proliferation and reverses the inhibition of cell proliferation caused by the downregulation of LINC01116 (Figure 4A,B). Flow cytometry suggested that the apoptotic rate of cells transfected with pcDNA3.1‐miR‐9‐5p or pcDNA3.1‐CCNE1 was lower than that of the NC group (***p < 0.001) (Figure 4C).

Western blot was used to analyse the expression of cell cycle‐related molecules (CCNE1 and CDK2), proliferation‐related markers (Ki‐67, PCNA and MCM7) and cell senescence markers (p53 and p16). The results showed that compared to the NC group, the expression of CCNE1, CDK2, Ki‐67, PCNA and MCM7 increased in the experimental group with miR‐9‐5p or CCNE1 overexpression, while that of p16 and p53 decreased, indicating that the overexpression of either of the molecule can reverse the changes in the protein level of LUAD cells caused by the downregulation of LINC01116 (Figure 4D).

### 3.5 LINC01116 Upregulates CCNE1 and Promotes the Proliferation of LUAD Cells by Binding to miR‐9‐5p

**FIGURE 5:**
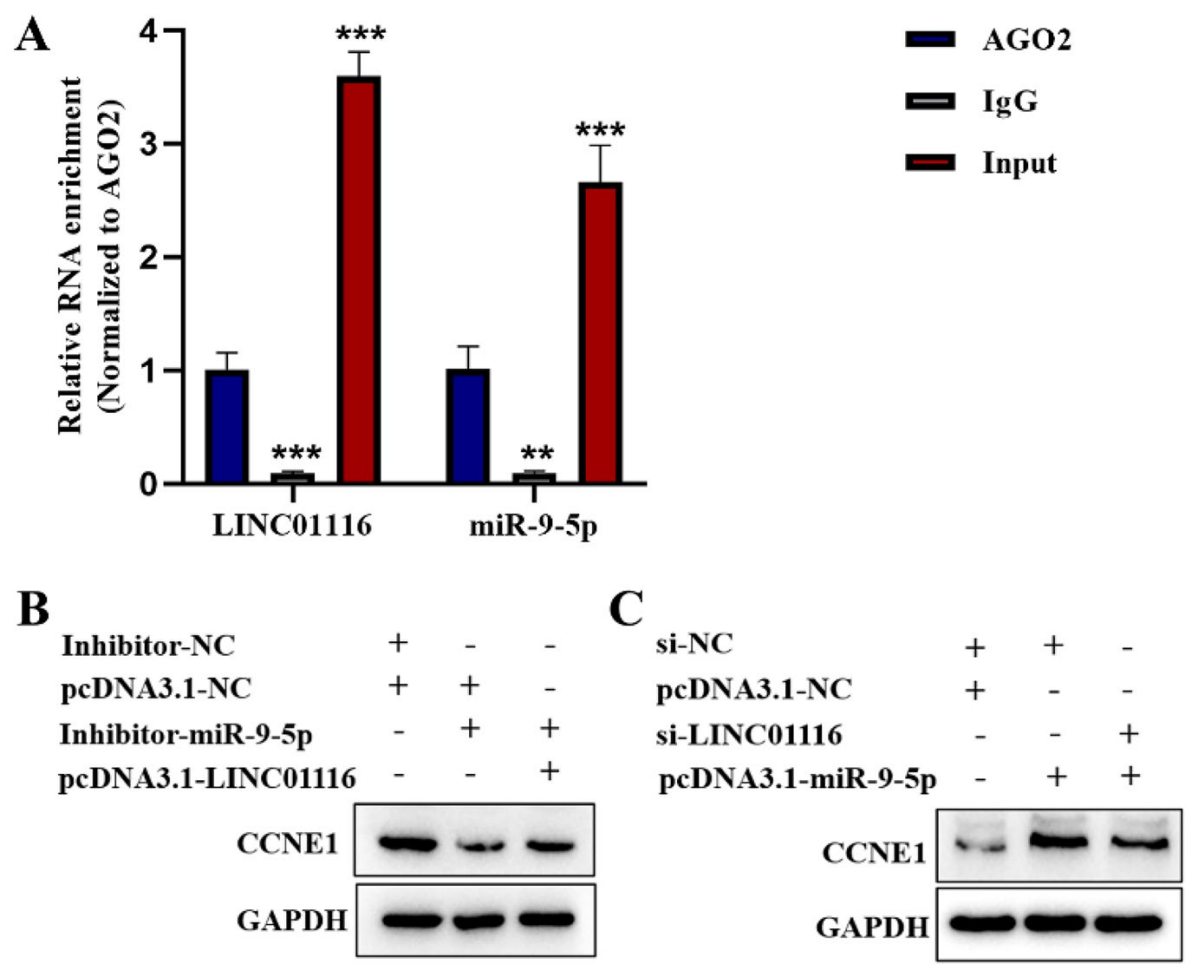
(A) qRT‐PCR based on RIP indicated that both LINC01116 and miR‐9‐5p can bind to miR‐744‐5p. One‐way ANOVA was applied for analysis (**p < 0.01 ***p < 0.001). (B) Protein level of CCNE1, regulated by LINC01116, was measured by the Western blot analysis. (C) Protein level of CCNE1, regulated by miR‐9‐5p, was estimated by the Western blot analysis.

Bioinformatics predicted the putative binding sites between miR‐9‐5p and LINC01116. The immunoprecipitation assays showed that the enrichment of LINC01116 and miR‐9‐5p was elevated by AGO2 (**p < 0.01 ***p < 0.001) (Figure 5A). Next, we then explored the interactions between CCNE1, LINC01116 and miR‐9‐5p by the Western blot. The results showed that downregulated miR‐9‐5p reduced the expression of CCNE1. And then overexpressing LINC01116 restored the level of CCNE1, indicating that LINC01116 increases CCNE1 expression (Figure 5B). Similarly, overexpressing miR‐9‐5p increased the CCNE1 expression. Downregulated LINC01116 subsequently restored CCNE1 levels, indicating that LINC01116 decreases CCNE1 expression (Figure 5C). This phenomenon confirmed a co‐directional relationship between miR‐9‐5p and CCNE1, as well as between LINC01116 and CCNE1.

### 3.6 Downregulation of LINC01116 Inhibits Cell Proliferation of Xenograft Tumour In Vivo, While Overexpression of miR‐9‐5p or CCNE1 Promotes Cell Proliferation of Xenograft Tumour In Vivo

**FIGURE 6:**
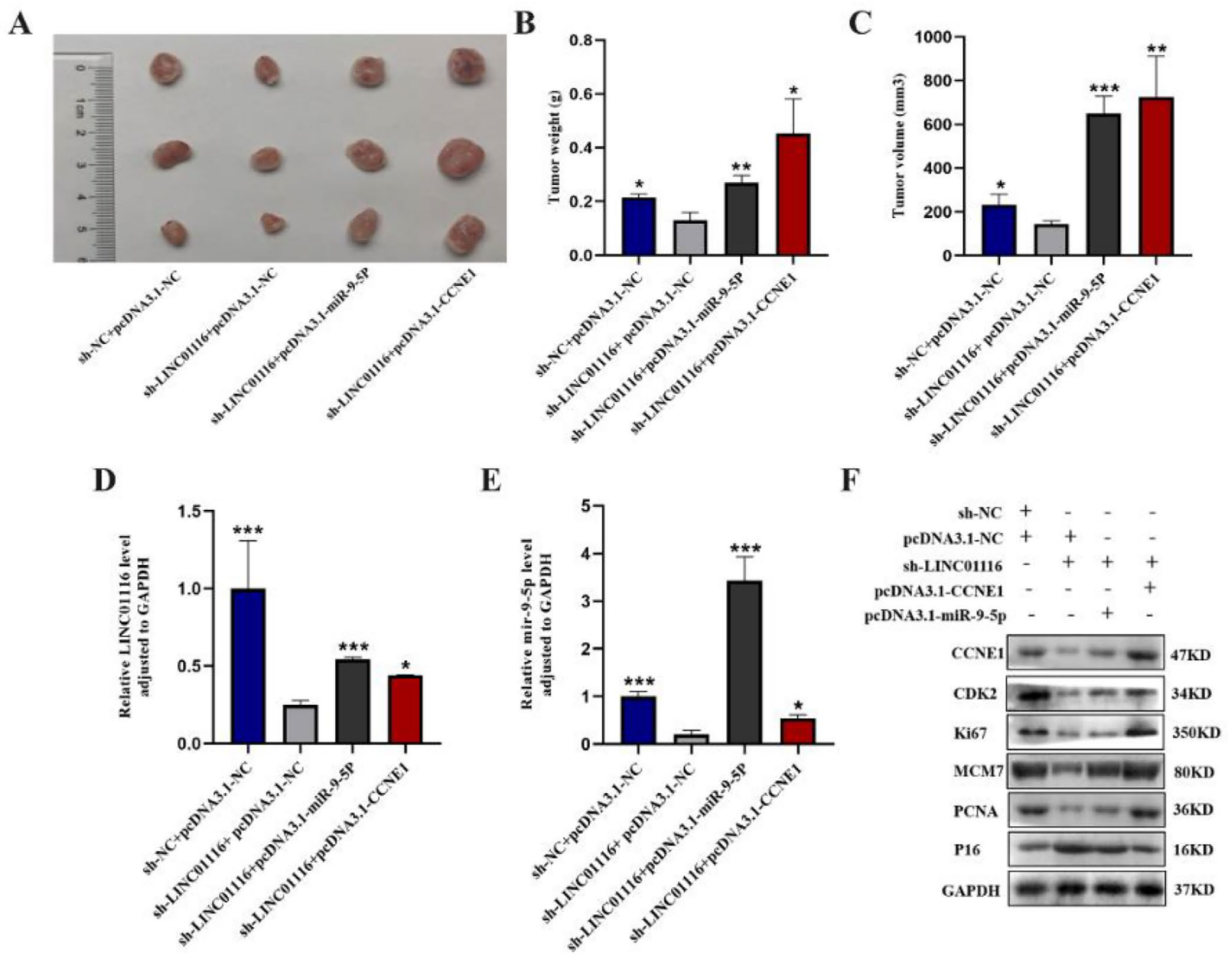
(A) The right panels: cells were transplanted with sh‐NC, sh‐LINC01116, pcDNA3.1‐ miR‐9‐5p or CCNE1 plasmids (*n* = 3). The left panels: the weight and volume of each tumour were measured (*p < 0.05, **p < 0.01, ***p < 0.001). (B) The tumour weight in the sh‐LINC01116 group was decreased, and the pcDNA3.1‐miR‐9‐5p/CCNE1 group was increased (*p < 0.05, **p < 0.01, ***p < 0.001). (C) The tumour volume was decreased in the sh‐LINC01116 group but increased in the pcDNA3.1‐miR‐9‐5p/CCNE1 group (*p < 0.05, **p < 0.01, ***p < 0.001). (D and E) The relative expression level of LINC01116 and miR‐9‐5p in the tumour tissue of BALB/C nude mice transplanted with sh‐NC, sh‐LINC01116, pcDNA3.1‐miR‐9‐5p or CCNE1 plasmids was achieved by qRT‐PCR (*p < 0.05, ***p < 0.001). (F) Protein expression of CCNE1, CDK2, Ki‐67 MCM7, PCNA and p16 in the tumour tissue of BALB/C nude mice, as measured by the Western blot analysis.

In order to test the oncogenic activity of LINC01116, miR‐9‐5p and CCNE1 in vivo, LUAD cells stably expressing sh‐LINC01116 and overexpressing miR‐9‐5p or CCNE1 stably were subcutaneously injected into the subcutaneous tissue of nude mice, establishing the xenograft tumour model. Compared to those injected with sh‐NC cells, tumour volume and weight were decreased in nude mice injected with sh‐LINC01116 and increased in nude mice injected with pcDNA3.1‐miR‐9‐5p or pcDNA3.1‐CCNE1 cells (*p < 0.05, **p < 0.01, ***p < 0.001) (Figure 6A–C).

Furthermore, in agreement with the in vitro results, qRT‐PCR confirmed the increased expression of LINC01116 and miR‐9‐5p (Figure 6D,E). Similarly, the results of the Western blot demonstrated that the expressions of LINC01116, miR‐9‐5p, CCNE1, CDK2, MCM7, Ki‐67 and PCNA were decreased, whereas that of p16 increased in the sh‐LINC01116 cells (vs. the NC group). In addition, the overexpression of miR‐9‐5p or CCNE1 attenuated sh‐LINC01116‐mediated inhibition of LINC01116, miR‐9‐5p, CCNE1, CDK2, MCM7, Ki‐67 and PCNA expression, while the disinhibition effect of pcDNA3.1‐CCNE1 was distinct (Figure 6F). These findings suggested that the carcinogenesis of LINC01116 in LUAD was mediated by miR‐9‐5p and CCNE1 in vivo.

### 3.7 LINC01116 and CCNE1 as Biomarkers for the Diagnosis and Prognosis of LUAD

Previous studies have reported that lncRNAs serve as prognostic biomarkers for various cancers [15, 16]. We performed the Kaplan–Meier analysis to determine whether the expression of LINC01116 and CCNE1 was related to the cancer‐specific survival rate in lung cancer patients.

**FIGURE 7:**
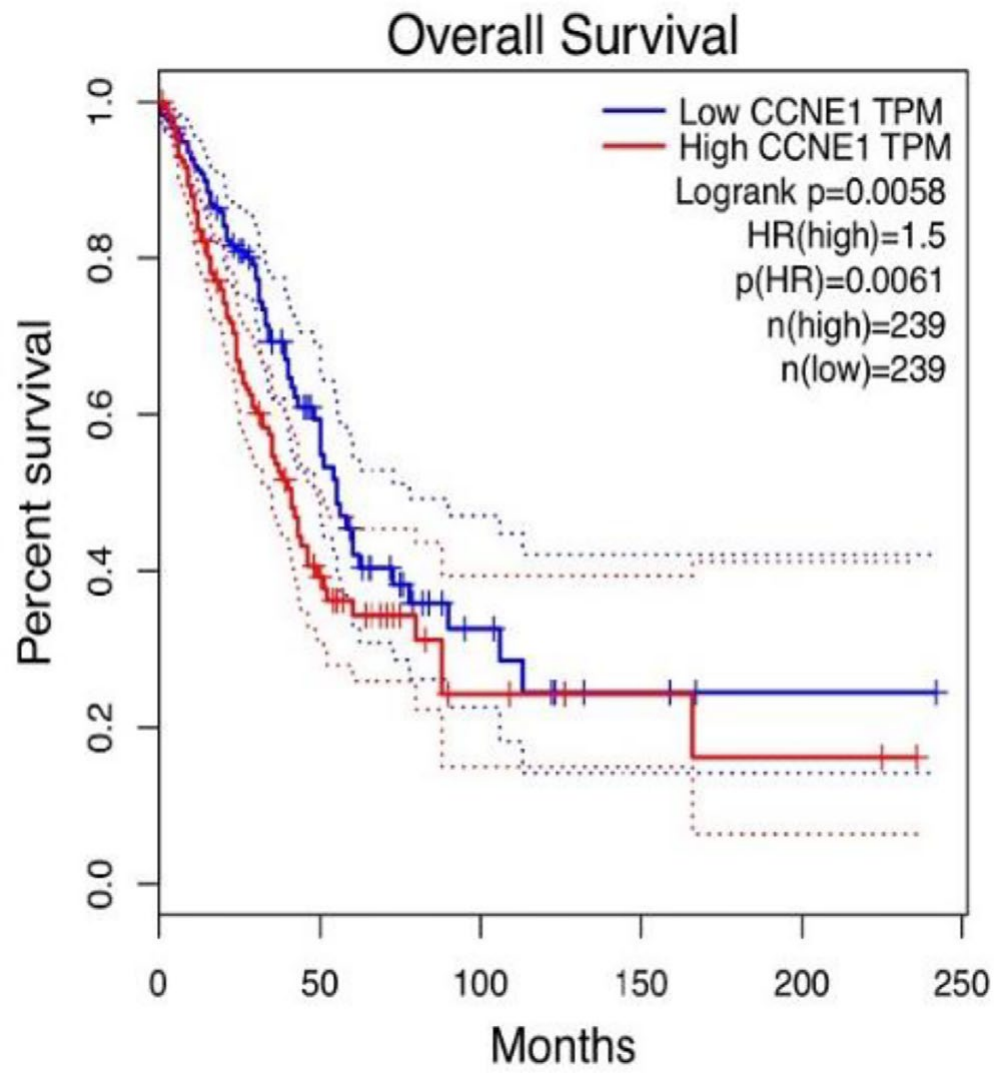
Kaplan–Meier survival analysis shows significantly different survival curves between LUAD patients with high and low expression levels of CCNE1 (*p* = 0.0058). The abscissa of the survival curve is the observation time, and the ordinate is the overall survival rate.

Taking the median expression level of LINC01116 in lung cancer as the cutoff point, the patients were divided into the high‐level (*n* = 239) and low‐level (*n* = 239) groups. The expression of LINC01116 was negatively correlated with tumour‐specific survival (*p* = 0.0019) (Figure 1Ea). Similarly, the expression of CCNE1 was negatively correlated with tumour‐specific survival (*p* = 0.0058) (Figure 7). These findings proved that LINC01116 and CCNE1 are effective predictors for the diagnosis of LUAD.

## 4 Discussion

Currently, lung cancer is one of the most common cancers worldwide. Histopathology is the main diagnostic basis of lung cancer that can provide satisfactory diagnostic accuracy, while due to various factors, such as sample size, different sampling sites and tumour heterogeneity, it cannot define the subtype accurately [17]. The curative effect of lung cancer is mainly due to the improvement in the early diagnosis technology and the early prediction of the curative effect of surgery, radiotherapy, chemotherapy and biotherapy. Tumour microenvironment (TME) can promote anti‐tumour immunity in SCLC [18], and the combination of immunotherapy targeting CSCs with standard drugs is also a promising direction [19]. Studies [20, 21] have found that biomarkers detected at the level of lncRNAs can be used for prognosis and immunotherapy guidance in lung cancer patients. Therefore, identifying tumour‐specific molecular markers and formulating individualised therapeutic strategies based on molecular classification would improve the curative effect of the complex treatment of lung cancer.

In recent years, several studies have shown that lncRNAs competitively bind miRNA through the ceRNA mechanism and regulate the related mRNA. This binding plays a crucial role in the occurrence and development of cancer in various systems, showing the feasibility of usage as a biomarker for clinical diagnosis and prognosis. Numerous studies have shown that lncRNAs, as key regulators of gene expression, play roles in tumour progression [22, 23, 24] Abnormal expression of lncRNAs has been implicated in the development of various diseases, including diabetic retinopathy [25]. For example, lncRNA THUMPD3‐AS1 has been shown to promote chondrocyte proliferation and enhance the inflammatory response [26]. In endometrial cancer, lncRNA NIFK‐AS1 inhibits the polarisation of macrophages by targeting miR‐146a, thereby inhibiting cell proliferation, migration and invasion and affecting its prognosis [27]. LncRNA NEAT1 regulates the progression of hepatocellular carcinoma (HCC) induced by chronic hepatitis C virus by regulating the miR‐9‐BGH3 axis [28]. In breast cancer, lncRNA Malat1 promotes cell proliferation, inhibits cell apoptosis and destroys the tumour immune microenvironment [29]. In addition, techniques such as HeadTailTransfer and MPCLCDA have significantly improved the accuracy of predicting ncRNA‐protein or lncRNA‐protein interactions [30, 31, 32], as well as circRNA‐disease associations [33], by using advanced sampling methods and metapathway selection. These computational advances have expanded the understanding of lncRNA interactions and supported the study of mechanisms such as LINC01116 and miR‐9‐5p/CCNE1 axis. LINC01116 was first found in prostate cancer, and gene knockdown reduces the integration ability of cancer cells and inhibits carcinogenesis [34]. A recent study showed that lncRNA LINC01116 is abnormally expressed in various cancers. According to the report of breast cancer, LINC01116 competitively combines with miR‐145 to regulate ESR1, which is a novel prognostic biomarker of breast cancer [35]. lncRNA LINC01116 can target miR‐520a‐3p and VEGFA and arrest cell proliferation and preclude brain and osteosarcoma tumorigenesis [36]. Additional studies have shown that LINC01116 is associated with chemotherapy resistance in certain cancers, particularly in response to treatments like gefitinib. Studies have shown that downregulation of LINC01116 can lead to gefitinib resistance, while its overexpression sensitises cells to gefitinib [37]. Thus, targeting LINC01116 could help reduce tumour resistance to treatment and enhance the efficacy of chemotherapeutic drugs. This study showed that LINC01116 is highly expressed in LUAD tissues, and its downregulation can inhibit the proliferation of LUAD cells and promote apoptosis.

Studies have demonstrated that the dysregulation of miRNAs is closely associated with disease progression. For example, miRNAs have been strongly linked to the progression of colorectal cancer [38]. Additionally, recent studies have identified that miRNA‐21 may serve as a potential biomarker for glioma, demonstrating high diagnostic accuracy [39]. The analysis of relevant databases confirmed that the targeted miRNA of LINC01116 is miR‐9‐5p. The miR‐9 family is one of the major members of miRNAs, with a critical role in promoting or inhibiting cancer through regulating the target genes in the tumorigenesis network. The expression of miR‐9 is upregulated in HCC as a cancer‐promoting gene and can specifically inhibit the expression of p21 gene and promote the progression of HCC. In glioma, upregulated miR‐9 inhibits the expression of COL18A1, THBS2, PTCH1 and PHD3, promoting tumorigenesis and angiogenesis [40, 41]. In recurrent ovarian cancer, the expression of miR‐9 is downregulated, acting as a putative tumour suppressor gene and serving as a potential biomarker [42]. Similarly, miR‐9‐5p belongs to the miR‐9 family and plays a major role in different types of tumours. Several studies have shown the increased expression level of LINC01116 in NSCLC, which promotes the proliferation and invasion of tumour cells [43, 44]; miR‐9‐5p was identified as the significant downstream gene. In the current study, LINC01116 binds to miR‐9‐5p, and the overexpression of miR‐9‐5p promotes the proliferation of LUAD cells and inhibits apoptosis.

An unlimited proliferation ability is crucial for tumorigenesis. A network of related proteins precisely controls cell proliferation, determining the development sequence of the cell cycle. The progression of cell cycle is mainly regulated by a family of CDKs. Cyclins form complexes with and are regulators of CDK kinases. It exhibits different expressions and degradation patterns at various stages of mitosis, coordinating cellular processes [45]. Cyclin E1 (CCNE1) forms a complex with and functions as a regulatory subunit of CDK2, whose activity is required for cell cycle G1/S transition and chromosome instability [46]. Reportedly, the abnormal regulation of CCNE1 is effective in cell proliferation, which is associated with poor prognosis and disturbance of tumour immune microenvironment [47, 48]. In the preceding preparation stage, we found that miR‐9‐5p binds to multiple target genes. KEGG enrichment analysis found that these genes were closely related to the cell cycle; hence, CCNE1 was selected as the target gene. Research on LINC01116 in cancer has highlighted its ability to bind to miRNAs, modulating target mRNA stability [8, 49]. Unlike prior studies that focus on single pathways, this study investigates a broader regulatory network in LUAD, examining both direct and indirect effects of miR‐9‐5p on CCNE1. In the current study, LINC01116 targeted miR‐9‐5p/CCNE1 axis and overexpression of CCNE1 significantly downregulates the protein changes induced by LINC01116, promoting the malignant biological behaviour of LUAD cells. Both LINC01116 and miR‐9‐5p positively regulated CCNE1. This integrative approach reveals the significant regulatory effects of LINC01116 on cell proliferation and apoptosis, positioning it as a key factor in maintaining LUAD oncogenicity. However, the regulatory mechanism of miR‐9‐5p is not yet clarified, and hence, we identified TGF‐β receptors (*TGFBRI* and *TGFBRII*) as putative candidates based on the literature review. The expression of TGFBRII and TGFB1 is lower in oesophageal adenocarcinoma (EAC) compared to normal oesophagus. In > 80% of Barrett's oesophagus (BE) and EAC samples, TGF‐β signalling is impaired, and genomic alterations in the members of the superfamily of TGF‐β pathway components occur in 65% of EAC. Interestingly, TP53 (encoding the tumour suppressor p53) and CDKN2A (encoding the cell cycle inhibitor p16) are frequently altered in BE and EAC. The combination of such driver mutations with the loss of function of members of the TGF‐β pathways may predispose BE to transition to cancer [50]. In the current study, the expression of p53 and p16 is decreased in the lung cancer of NSCLC compared to the normal tissue. Simultaneously, we found that miR‐9‐5p directly targets tumour suppressor gene *TGFBRII*; its overexpression negatively regulates this receptor [51]. Combined with the previous findings, this mechanism might promote the occurrence and development of lung cancer. We also identified a protein interaction between CCNE1 and TGFBRI through the database. Type II receptors phosphorylate type I receptors and activate their kinase activity [52]. The overexpression of miR‐9‐5p may affect the activity of TGFBRI while inhibiting TGFBRII translation, thereby increasing the expression of CCNE1 and offsetting the inhibitory effect of the overexpression of miR‐9‐5p on CCNE1. This phenomenon supplements the theory that lncRNA competes with mRNA. In addition, tumour‐related signalling pathway factors for miRNA binding BGH3 levels are also upregulated in HCV‐induced HCC and TCGA tissue samples, which could be directly correlated with NEAT1 levels.

Cell sequencing demonstrated that the expression of CDK6 was significantly downregulated in the samples transfected with si‐LINC01116. Western blot assay confirmed that p16, a member of CDKI‐INK4 family, was significantly upregulated in LUAD cells with the knockdown LINC01116 gene. In the treatment of oestrogen‐positive breast cancer, the endogenous inhibitor p16 binds to and inhibits the cyclinD‐CDK4/6 complex, but acquired resistance emerges immediately, which greatly reduces the effectiveness of monotherapy. Some studies have shown that the combination of p16 and PI3K inhibitors leads to sustained growth stagnation and also increases cell apoptosis and tumour regression in vivo [53]. Several studies have demonstrated that CDK4/6 inhibitors are also significant for squamous cell NSCLC [54]. Due to the amplification of acquired CCNE1 in breast and pancreatic cancers, the cells have acquired resistance to CDK4/6 inhibitors in vitro, damaging the sensitivity of cells to CDK4/6 inhibitors but still rendering them sensitive to CDK2 inhibitors [53, 55]. In our study, CCNE1 was amplified in LUAD cells with a high expression of LINC01116. Hence, the combination of CCNE1 and CDK2 inhibitors needs to be investigated further. Our findings suggest that LINC01116 could be a promising target for LUAD diagnosis and treatment, enriching our understanding of ceRNA networks and offering a potential new avenue for therapy through the LINC01116/miR‐9‐5p/CCNE1 axis.

## 5 Conclusion

In summary, we found that LINC01116 promotes the proliferation and apoptosis of LUAD cells by positively regulating the miR‐9‐5p/CCNE1 axis. This finding provides a putative for the treatment of LUAD and also supplements the ceRNA theory.

## Author Contributions

**Hui Zhang:** data curation (equal), investigation (equal), validation (equal), writing – original draft (equal), writing – review and editing (equal). **Wenwen Cai:** data curation (equal), investigation (equal), validation (equal), writing – original draft (equal), writing – review and editing (equal). **Yiyan Miao:** data curation (supporting), investigation (supporting), validation (supporting), writing – original draft (supporting). **Yihang Gu:** formal analysis (equal). **Xiaorong Zhou:** conceptualization (equal), funding acquisition (equal), methodology (equal), supervision (equal). **Hiroyasu Kaneda:** supervision (equal). **Lan Wang:** conceptualization (equal), funding acquisition (equal), methodology (equal), supervision (equal).

## Ethics Statement

Ethics approval was obtained from the Research Ethics Committees of the Laboratory Animal Center of Nantong University with Ethical approval number; P20221223‐005. All experiments were performed in accordance with relevant guidelines and regulations.

## Consent

Informed consent was obtained from all the donors prior to the collection of samples.

## Conflicts of Interest

The authors declare no conflicts of interest.

## Data Availability

The data that support the findings of this study are available from the corresponding author upon reasonable request.
